# Beyond the known phenotype of sotos syndrome: a 31-individuals cohort study

**DOI:** 10.3389/fped.2023.1184529

**Published:** 2023-06-13

**Authors:** Lourdes Vega-Hanna, Mario Sanz-Cuesta, Didac Casas-Alba, Mercè Bolasell, Loreto Martorell, Leticia Pías, Ana Lucia Feller, Antonio Federico Martínez-Monseny, Mercedes Serrano

**Affiliations:** ^1^ Department of Pediatrics, Hospital Sant Joan de Déu Barcelona, Barcelona, Spain; ^2^ Department of Pediatrics, Hospital de Sant Boi, Parc Sanitari Sant Joan de Déu, Barcelona, Spain; ^3^ Department of Genetic and Molecular Medicine/IPER, Institut de Recerca, Hospital Sant Joan de Déu Barcelona, Barcelona, Spain; ^4^ Pediatric Neurology Department, Institut de Recerca, Hospital Sant Joan de Déu, Barcelona, Spain; ^5^ Departamen of Pediatrics, Hospital J P Garrahan, Buenos Aires, Argentine; ^6^ Centro de Investigación Biomédica en Red de Enfermedades Raras (CIBERER), Instituto de Salud Carlos III, Barcelona, Spain

**Keywords:** sotos syndrome, phenotype, overgrowth, *NSD1*, *NFIX*, *APC2*

## Abstract

**Introduction:**

Sotos Syndrome (SS, OMIM#117550) is a heterogeneous genetic condition, recognized by three main clinical features present in most cases: overgrowth with macrocephaly, typical facial appearance and different degrees of intellectual disability. Three different types are described caused by variants or deletions/duplications in *NSD1, NFIX* and *APC2* genes. We aimed to describe a cohort of pediatric patients reporting the typical and unexpected findings in order to expand the phenotype of this syndrome and trying to find genotype-phenotype correlations.

**Methods:**

In our referral center, we collected and analyzed clinical and genetic data of 31-patients cohort diagnosed with SS.

**Results:**

All of them presented with overgrowth, typical dysmorphic features and different degree of developmental delay. Although structural cardiac defects have been reported in SS, non-structural diseases such as pericarditis were outstanding in our cohort. Moreover, we described here novel oncological malignancies not previously linked to SS such as splenic hamartoma, retinal melanocytoma and acute lymphocytic leukemia. Finally, five patients suffered from recurrent onychocryptosis that required surgical procedures, as an unreported prevalent medical condition.

**Discussion:**

This is the first study focusing on multiple atypical symptoms in SS at the time that revisits the spectrum of clinical and molecular basis of this heterogeneous entity trying to unravel a genotype-phenotype correlation.

## 1. Introduction

Sotos Syndrome (SS, OMIM#117550) is a heterogeneous genetic condition, recognized by three main clinical features present in most cases: overgrowth, defined as height and/or head circumference at least two standard deviations above the mean; typical facial appearance and different degrees of intellectual disability. Indeed, SS is the most frequent genetic cause of overgrowth, with an estimated incidence of 1/14.000 live births (1–3).

More than 90% of the patients present an autosomal dominant deletion or variant in the Nuclear Receptor SET Domain–containing protein 1 (*NSD1*) gene, located at chromosome 5q35, encodes a histone methyltransferase that catalyzes the transfer of methyl groups to lysine residues of histone tails: more specifically lysine residue 36 of histone H3 (H3K36) and less frequently lysine residue 20 of histone H4 (H4K20) (4). These methylation marks are most frequently associated with transcriptional activation but can be associated with repression depending on the cellular context (5). Loss of function experiments in animal models revealed that *NSD1* is essential for normal development and has been confirmed to also play an important role in human developmental syndromes, such as SS, as well as in different types of malignancies (6–10).

Other causes may correspond to variants in the recently described *NFIX* or *APC2* genes (located on chromosome 19p13) that explain SS type two (also called Malan syndrome) and SS type three (11–13). There are also other syndromes characterized by overgrowth, mainly Tatton-Brown-Rahman Syndrome, Weaver Syndrome, or Sotos-like Syndrome, caused by variants in *DNMT3A*, *EZH2,* and *SETD2* genes, respectively (14, 15).

The typical overgrowth pattern of SS starts prenatally, where patients may have higher mean birth length, weight, and head circumference, but contrarily, many reach adulthood with a height within the upper normal range. Hypotonia, feeding difficulties, hypoglycemia, and neonatal jaundice are also commonly found during the perinatal period (16–18).

SS dysmorphic features include macrocephaly and dolichocephaly, frontal bossing, high anterior hairline, down-slanted palpebral fissures, high arched palate, pointed and triangular chin, and large hands and feet. Clinically, SS patients may present neurodevelopmental delays associated with learning and behavior problems, attention deficit with hyperactivity, and socialization disturbances with autistic traits (19–23). Other complications include epilepsy, impaired vision and hearing, cardiac, urinary, and orthopedic defects, recurrent infections, and increased oncological risk (5, 24).

In this work, we aimed to describe a cohort of pediatric patients diagnosed with SS, focusing on the unreported medical complications, and expanding the clinical and genetic profile of the syndrome. We reevaluate the possibility of un-described phenotype-genotype correlations.

## 2. Materials and methods

### 2.1. Patient cohort

We conducted a descriptive, observational, and ambispective study. We included patients according to the following criteria: pediatric patients (between 2 and 18 years at the time of diagnosis) with a confirmed molecular diagnosis of SS attended at a tertiary hospital [Hospital Sant Joan de Déu (HSJD), Barcelona Spain] between January 2012 and July 2020.

We analyzed the medical records of a total of 31 pediatric patients. Information on clinical characteristics and complementary exams was analyzed. Adolescence was considered between 10 and 19 years of age, according to the World Health Organization definition.

Parents or legal representatives gave their written informed consent, and children/adolescents gave their assent. Blood samples were obtained in accordance with the Declaration of Helsinki revised in 2013.

### 2.2. Genetic analyses

In most patients (particularly the older individuals who underwent previous study protocols), genetic screening was performed using either Multiple Ligation Probe Assay (MLPA) or Array-CGH to search Deletions/Duplications of *NSD1* and *NFIX* genes. In many patients (with negative MLPA/Array-CGH or those more recently enrolled) Sanger studies or clinical exome sequencing and subsequent confirmation and segregation of variants with Sanger sequencing was performed. All variants resulted *de novo*.

SALSA MLPA Probemix P026-E2 Sotos (MRC-Holland) has one probe for each one of the 23 exons from *NSD1* and the 10 exons of *NFIX* genes and allowed us to detect any alteration at the exon level.

Array-CGH analysis was performed using a comparative genomic hybridization oligonucleotide microarray (qChipCM, 8 × 60 K; qGenomics). The submitted sample was hybridized against a commercial reference DNA of the same sex (Agilent Technologies). The quality of the data obtained was evaluated following the manufacturer's recommendations.

Primers used for sanger sequencing were designed with Primer3 program (httP://bioinfo.ut.ee/primer3/ analysis was performed by comparison with the reference sequence for *NSD1* (NM_0022455.5).

Exome sequencing of the coding regions (exons, and 25 bp intronic regions flanking the exon) of 6,710 genes associated with pathology according to the Human Gene Mutation Database (HGMD), GeneTest.org, and the Online Mendelian Inheritance in Man catalog (OMIM) was performed. Next-generation sequencing (NGS) techniques were employed using enrichment by hybridization in solution with an Illumina design kit (TruSight One Sequencing Panel) and subsequent sequencing on an Illumina NextSeq500 sequencer. The bioinformatic analysis for the exome data was carried out using a pipeline developed in the bioinformatics unit within our hospital.

### 2.3. Clinical follow up

Once the SS diagnosis is established, the follow up of these patients consists of a once per year visit in a multidisciplinary visit including genetics and neuropediatrics. During these follow-ups, a detailed physical examination is performed, which includes anthropometric measurements, dysmorphologic descriptions, and back examinations for scoliosis. Additionally, endocrinology visits are appointed biannually, and oncological assessments are performed by specialists when the genetic diagnostic is confirmed. All the families have the contact of a patient manager, in case any health complication or necessity appears.

Since other comorbidities need to be ruled out at the time of diagnosis and during the follow up, an echocardiogram and renal ultrasound tests are routinely performed. Individuals are also systematically, referred for an audiology assessment.

### 2.4. Statistical analyses

Descriptive analyses were performed using univariate (Chi squared tests corrected by Pearson and Fisher's exact tests). Two-sided tests yielding a *p* < 0.05 was considered statistically significant. SPSS statistics for Windows, version 23.0 (IBM Corp., Armonk, NY, USA) was used to perform all statistical analysis.

### 2.5. Ethical approval

The study protocol was reviewed and approved by the Research & Ethics Committee of the Hospital Sant Joan de Déu, Barcelona, Spain (Project Internal Code PIC-08-19). The study was conducted in accordance with the Declaration of Helsinki, Good Clinical Practices, and applicable regulatory requirements. All parents and adult patients provided written informed consent, and adolescent patients able to understand the procedure gave their assent prior to patient enrollment.

## 3. Results

We analyzed 31 patients (16 males and 15 females) between 2 and 25 years of age (12.9 ± 5.9) in the last evaluation. The mean age at diagnosis was 8.0 (±5.9) years with a mean follow up time of 8.8 (±5.3) years. The clinical data of our cohort is described in Table 1 and Supplementary Table S1.

**Table 1:** Common features and other features not found in literature.

Common and previously reported features	Number (% of total)	Other features not found formerly in literature	Number (% of total)
Perinatal			
Hypotonia	11 (36)	Ovarian cysts (oophorectomy)	1 (3)
Neonatal Jaundice	11 (36)		
High weight or height (>4 kg or >52 cm)	9 (41)		
Prematurity (moderate)	7 (26)		
Respiratory distress syndrome	2 (7)		
Neurological			
Neurodevelopmental delay	24 (77)	Chronic mixed headache	3 (10)
Epilepsy	14 (45)		
ADHD	8 (26)		
Autism Spectrum Disorders	4 (13)		
Febrile seizures	4 (13)		
Neuroimaging^a^			
Ventricular dilation	13 (54)	Chiari malformation type 1	1 (4)
Hypoplasia or agenesis of corpus callosum	8 (33)	Septum pellucidum cyst	1 (4)
Subcortical WM lesions	4 (17)	Epidural Hematoma	1 (4)
Germinolitic cysts	3 (13)		
Cortical dysplasia	2 (8)		
Heart disease			
Atrial septal defect	4 (13)	Recurrent pericarditis	2 (7)
Aortic valve regurgitation	2 (7)	Ventricular preexcitation	1 (3)
Mitral valve prolapse	2 (7)	Cor triatriatum Dexter	1 (3)
Persistent ductus arteriosus	1 (3)	Left ventricular hypertrophy	1 (3)
		Aortopulmonary fistula	1 (3)
Ophthalmological			
Strabismus	8 (26)	Cataract (bilateral)	3 (10)
Hyperopia	6 (19)	Retinal melanocytoma	1 (3)
Congenital nystagmus	4 (13)	Pseudopapiledema	1 (3)
Astigmatism	3 (10)	Optic nerve	1 (3)
Retinal atrophy	3 (10)	Hypoplasia	
Skeletal			
Scoliosis	11 (36)	Onychocryptosis	5 (16)
Pes valgus	7 (23)	Craniosynostosis	2 (7)
Otolaryngological			
Adenoid and tonsilar hypertrophy	6 (19)	Turbinate hypertrophy	1 (3)
Chronic otitis media	6 (19)	Orbitary cellulitis	1 (3)
Hearing loss (conductive)	5 (16)		
Obstructive sleep apnea	4 (13)		
Dental			
Multiple caries disease	9 (29)		
Early dental eruption	3 (10)		
Dental agenesia	2 (7)		
Gastroenterological			
Gastroesophageal reflux (severe)	4 (13)	Gastric volvulus	1 (3)
Constipation (severe)	2 (7)	Cholelithiasis (asymptomatic)	1 (3)
		Celiac disease	1 (3)
Genitourinary			
Vesicoureteral reflux	3 (10)	Renal lithiasis	2 (7)
Duplicated collecting system	3 (10)		
Recurrent urinary infections	2 (7)		
Urinary tract dilation (high risk)	1 (3)		
Pneumological and immunological			
Asthma (persistent or (intermittent)	7 (23)	Cilia immotile syndrome	1 (3)
Pneumoallergen sensitivity	3 (10)	Splenic hamartoma	1 (3)
Food allergies	1 (3)	Impaired B cell maturation	1 (3)
Recurrent pneumonia	1 (3)		

^a^ Neuroimaging was performed in 24 patients.

### 3.1. Molecular findings

**Figure 1:**
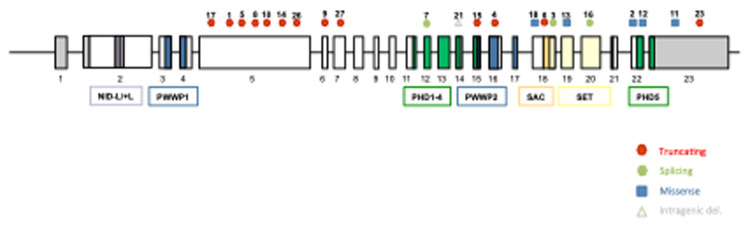
Schematic representation of *NSD1* gene, showing the localization of the variants described in our cohort. All 23 exons of *NSD1* are presented by boxes and introns are represented by lines. The domains of *NSD1* are represented with different colored boxes. *NSD1* contain two nuclear receptor interacting domains (NID), two proline-tryptophan-tryptophan-proline (PWWP), five plant homeodomain zinc fingers (PHD), the catalytic Su(var)3–9, enhancer-of-zeste, Trithorax (SET) and the C-terminal C5HCH (Cys-His) domain (SAC). Missense variants are represented with blue squares, truncating variants with red circles, intragenic deletions with yellow triangles and splicing variants with green circles.

Out of all patients, 27 (87%) corresponded to variants of the *NSD1* gene, with 18 point variants (5 missense and 13 truncating), 7 deletions, and 2 duplications (Figure 1). There were 4 patients who presented variants of the *NFX1* gene, with 2 missense variants, 1 deletion, and 1 duplication. The description of the genetic variants identified in each patient are stated in Table 2.

**Table 2:** Description of the genetic variants identified in each patient.

Patient	Variants
1	*NSD1 (NM_022455.5):c.1810C > T (p.Arg604Ter)*
2	*NSD1 (NM_022455.5):c.6364T > C (p.Phe2122Leu)*
3	*NSD1 (NM_022455.5):c.5892 + 1G > C*
4	*NSD1 (NM_022455.5):c.5332C > T (p.Arg1778Ter)*
5	*NSD1 (NM_022455.5):c.1973_1974insATCA (p.Asp659SerfsTer2)*
6	*NSD1 (NM_022455.5):c.5730dup (p.Cys1911MetfsTer9)*
7	*NSD1 (NM_022455.5) c.4765 + 2T > G*
8	*NSD1 (NM_022455.5):c.2362C > T (p.Arg788Ter)*
9	*NSD1 (NM_022455.5):c.3811A > T (p.Lys1271Ter)*
10	*NSD1 (NM_022455.5):*c.1870InsT (p.Ile623Ter)
11	*NSD1 (NM_022455.5):c.6558T > G (p.His2186Gln)*
12	*NSD1 (NM_022455.5):c.6377A > T (p.Asp2126Val)*
13	*NSD1 (NM_022455.5):c.5920G > A (p.Glu1974Lys)*
14	*NSD1* (NM_022455.5):c.2276C > G (p.Ser759Ter)
15	*NSD1* (NM_022455.5):c.5229G > A (p.Trp1743Ter)
16	*NSD1* (NM_022455.5):c.6010-1G > A
17	*NSD1 (NM_022455.5)*:c.1243C > T (p.Gln415Ter)
18	*NSD1* (NM_022455.5):c.5892G > T (p.Lys1964Asn)
19	*NSD1* deletion^a^
20	*NSD1* deletion^a^
21	*NSD1* (NM_022455.5):c.5079delTTACCCT (p.Phe1693Ter)
22	*NSD1* deletion^a^
23	*NSD1* (NM_022455.5):c.7485delA (p.Gly2496ValfsTer82)
24	*NSD1* deletion^a^
25	*NSD1* deletion^a^
26	*NSD1 (NM_022455.5):*c.2859dupT (p.Lys954Ter)
27	*NSD1* (NM_022455.5):c.4035dupT (p.Glu1346Ter)
28	*NFIX (NM_001271043.2):c.554T > C (p.Leu185Pro)*
29	*NFIX* (NM_001271043.2):c.276delG (p.Glu94ter)
30	*NFIX (NM_001271043.2):* c.367C > T (p.Arg123Trp)
31	NFIX (ENST00000676441.1):c.67_73dup (p.Glu25ValfsTer31)

^a^ By SALSA MLPA Probemix P026-E2 Sotos (MRC-Holland).

### 3.2. Perinatal characteristics

Hypotonia and neonatal jaundice were observed in a third of the individuals, showing a significant difference in those premature patients (*p* = 0.049 and *p* = 0.003 respectively). Only 12.9% presented feeding difficulties, being more prevalent in females (*p* = 0.043) and in patients with *NFIX* variants (*p* = 0.003). Seven subjects were born moderately preterm (Table 1).

### 3.3. Growth and characteristic facial appearance

High birth length (>52 cm) and weight (>4 Kg) was observed in 40.9% of the patients. A pronounced subsequent postnatal growth (at least 2 SD above the mean) was maintained in almost half of the patients. Macrocephaly (at least 2 SD above the mean) was present in 50% of the patients.

A characteristic facial appearance consisting of a high and broad forehead, sparse frontotemporal hair, downslanted palpebral fissures, malar flushing, and a pointed chin was present in all patients. Three patients presented microretrognathia and one patient presented facial asymmetry.

### 3.4. Developmental and neurological disorders

Psychomotor delay and a varying range of intellectual disability were observed in most patients (77.4%). As neurological comorbidities, epileptic seizures were found in almost half of the sample (45.2%), of which nine patients required medical treatment: valproic acid (VPA) in three patients; levetiracetam (LEV) and topiramate (TPM) both in one patient each; and associations of VPA plus LEV and VPA plus vigabatrin (VGB), both in two patients, respectively. Two patients suffered from simple febrile seizures, not followed by epilepsy.

In terms of behavioral disorders, attention-deficit hyperactivity disorder (ADHD) was diagnosed in eight patients (25.8%) and socialization difficulties were evident in seven patients (22.6%), including four with severe autism spectrum disorder (ASD) (12.9%). This last diagnosis was more prevalent in male patients (*p* = 0.05).

Meanwhile, other comorbidities such as impulsive behavior and anxiety disorder were diagnosed in 11 patients (35.5%), being the latter more prevalent in teenagers and young adults (*p* = 0.01).

No genetic correlations were identified between variants, affected genes, and the presence of epilepsy or behavioral disorders.

### 3.5. Heart disease

Regarding heart disease, 13 patients (41.9%) were diagnosed with some form of it. Cardiac structural defects were the most frequent (38.7%) (Table 1), and patients with hypotonia at birth were at increased risk of developing them (*p* = 0.042). These included atrial septal defects and persistent ductus arteriosus, observed in four patients, one of which was a preterm baby. Other findings included the diagnosis of mitral valve prolapse, *cor triatriatum dexter,* and left ventricle hypertrophy.

**Figure 2:**
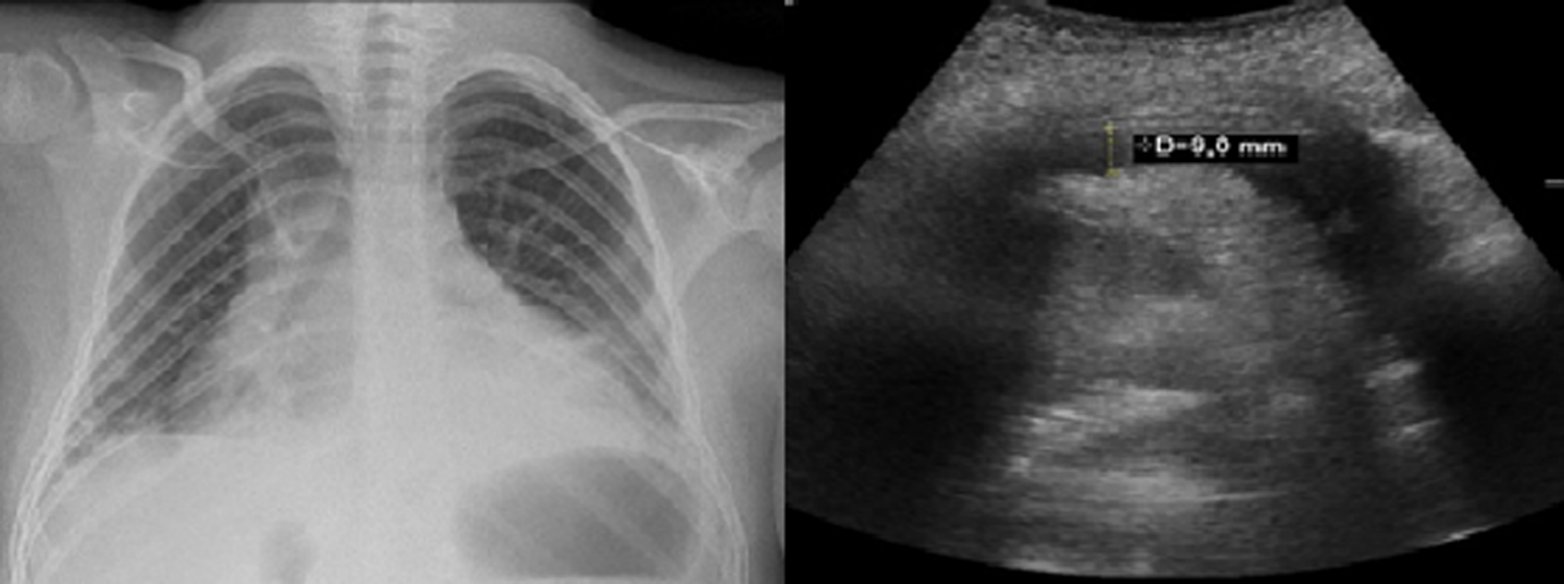
Pericardial effusion. **Left**: Thorax radiography showing cardiomegaly. **Right**: echocardiogram of patient with pericardial effusion of 9 mm.

Two patients presented recurrent pericarditis with pericardial effusion (Figure 2). Clinical presentation started with a viral infection as a trigger leading to pericarditis without positive evidence of infectious or rheumatic etiology. These patients underwent multiple relapses and ended up depending on chronic immunosuppressant therapy (corticosteroid therapy). Both cases of pericarditis were detected in patients with *NSD1* affectations. One of them presented an *NSD1* deletion, and the other one a nonsense variant in the same gene *NSD1*:c.3811A > T (p.Lys1271Ter).

Arrhythmia was detected in one patient, which was further diagnosed with ventricular preexcitation without paroxysmal tachycardia. There was no statistically significant correlation between the presence of heart disease (structural or not) and molecular findings.

### 3.6. Ophthalmological disorders

Twenty-two patients (71%) presented ophthalmological disorders, including strabismus (eight patients) and refractive defects (hyperopia in six patients and astigmatism in three patients) as the most prevalent. Congenital nystagmus affected four patients. Moreover, three patients showed bilateral cataracts that needed surgery during the first months of life, and three developed retinal atrophy. One subject presented a retinal melanocytoma, explained in the oncological findings section.

### 3.7. Otolaryngological disease

Chronic otitis media leading to conductive hearing loss and adenoid and tonsil hypertrophy were the most prevalent diseases, both in six patients. Four of the subjects suffered from apnea-hypopnea syndrome, due to adenoid and tonsil hypertrophy or microretrognathia, plus the hypotonic muscle tone. Two patients presented cholesteatoma, one of them associated with hypoacusis. Both patients required surgical intervention.

### 3.8. Traumatological and orthopedic disorders

The most frequent skeletal finding in our cohort was scoliosis, present in 11 patients (35.5%), two of them required surgical intervention. Pes valgus was observed in seven patients (22.6%). Five patients (16.1%) suffered from recurrent onychocryptosis needing surgical procedures due to the lack of improvement with conservative treatment.

### 3.9. Genitourinary and endocrinological findings

Eight patients (25.8%) suffered from genitourinary pathology including urinary tract dilation, duplicated collecting system, renal dysplasia/hypoplasia, or renal agenesis. Unilateral cryptorchidism was reported in two patients. One child suffered from hydrocele testicle and needed surgical treatment. One boy presented idiopathic precocious puberty without abnormalities at the sellar and suprasellar regions in neuroimaging.

### 3.10. Oncological findings

**Figure 3:**
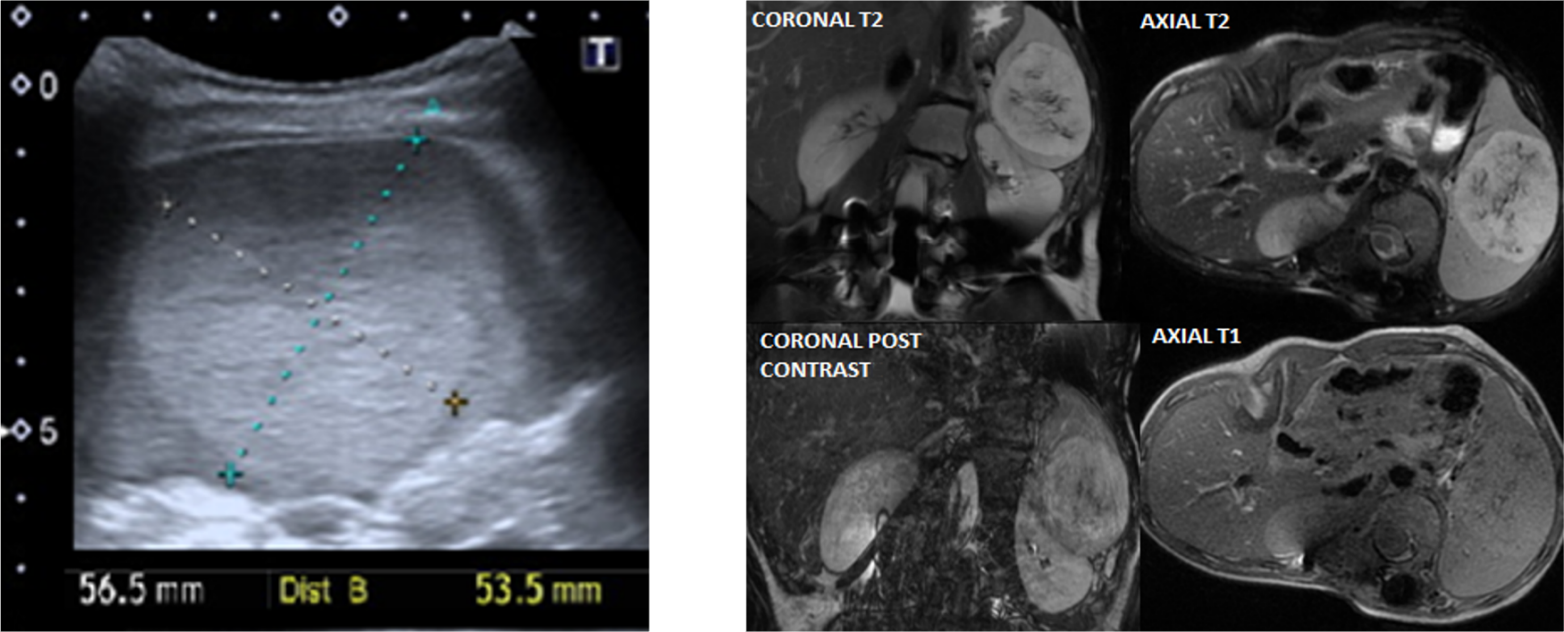
Splenic hamartoma clinical imaging. **Left** panel: Ultrasound of a patient with well-defined splenic solid mass, 5.7 × 5.4 centimeters, isoechoic, with calcifications dotted inside. **Right** panel: MRI imaging of the same patient that shows stable mass of 7.2 × 5.9 × 6.9 cm, hyperintense in T2, isointense in T1, without fat, with enhancement in small hemorrhagic areas.

Within our cohort, we identified three different oncological presentations. One case of splenic hamartoma (Figure 3), diagnosed at seven years of age, in a routine study that remained stable in subsequent controls; one case of retinal melanocytoma, diagnosed at one year of age, showing spontaneous involution; and one case of acute lymphocytic leukemia diagnosed at 14 years of age, to whom chemotherapy treatment is currently ongoing. Missense variants in the *NSD1* gene were drivers of increased oncological malignancies compared to *NFIX* alterations (*p* = 0.049) in our cohort.

## 4. Discussion

SS is a rare genetic disorder characterized by a range of features, which have mainly been described in adult populations and small pediatric cohorts with overgrowth syndromes. In this study, we aim to provide a comprehensive description of the typical and atypical findings in a large cohort of pediatric patients with SS, all followed in the same center.

Regarding the conventional and extensively documented observations, the prevailing feature observed in all patients was the distinct facial appearance. While macrocephaly is commonly regarded as a hallmark symptom of SS, it was only detected in half of our patient cohort. Nonetheless, the particular skull shape was also observed in the non-macrocephalic subjects, pointing towards SS as a potential diagnosis.

There were no specific remarkable perinatal findings. Mild prematurity, neonatal jaundice, and perinatal global hypotonia are frequently found in SS individuals and may rise the suspicion of a genetic condition. Contrarily, hypoglycemia was not as frequent as expected.

As for neurological assessment, neurodevelopment delay was present in 77.4% of the sample. Classically, SS patients reported in the literature always presented with intellectual disability. For example, some studies found that 5q35 microdeletions are linked with lower scores in cognitive, adaptive functioning, and behavioral domains (25). However, other studies are starting to show a varying range of cognitive ability and some patients can be found within a normal cognitive range (18, 21).

In our sample, the range of intellectual difficulties is also wide, with many patients attending normal schooling with some subject adaptations. ADHD has been reported as a very prevalent neurodevelopmental disorder in this population (19) and probably is underdiagnosed in our cohort since the coexistence of intellectual disability and ADHD traits may complicate the diagnosis.

The present study reveals a notable prevalence of ASD traits in our sample, despite not meeting all of the classical criteria for ASD. This finding is consistent with previous reports in the literature (19–22). Again, the coexistence of a typical behavioral phenotype with traits of other neurodevelopmental conditions (perhaps not fulfilling the classical criteria), complicates the diagnosis. However, particular recognition and correct management of ADHD and ASD may have a relevant impact on the patient's development, including academic achievements, social inclusion, but also the quality of their home life.

Regarding behavioral issues, tantrums and anxiety were found to be the most prevalent. The behavioral disturbances observed may be attributed, in part, to the characteristics of ADHD and ASD, such as impulsiveness, rigidity, and social difficulties. In view of our results and the impact on patients' and families' wellbeing, guidelines need to include mental health experts (both psychologists and psychiatrists) implied in the assessment and follow-up of this group of patients. Regarding epilepsy, in concordance with the literature (5, 26), presented in half of our patients and was well controlled with antiepileptic drugs, mostly in monotherapy. None of the patients presented neurological regression due to uncontrolled epilepsy. The presence of febrile seizures was not related to subsequent epilepsy.

In our study, we wanted to take a holistic view of SS by considering a wide array of comorbidities and make an exhaustive analysis to expand the clinical phenotype. Subsequently, we have identified a series of characteristics linked to SS in our cohort, rarely described in the literature. One such characteristic is the presence of recurrent pericarditis or arrhythmias.

According to established studies, between 20% and 50% of SS patients may present with cardiac defects (27), but these usually include septal or valvular defects.

In our cohort, two patients exhibited recurring pericarditis, which manifested at comparable ages (nine and ten years old) and with similar clinical presentations (associated with viral infection triggers, multiple relapses, and dependence on corticosteroid treatment). Despite the patients' similar presentations, infectious and rheumatologic etiologies were ruled out. Recently, one study reported pericarditis in a patient with SS, which was observed in the context of SARS-CoV-2 infection, highlighting SS as a potential risk factor for this condition during viral infections (28). Therefore, the possibility of recurrent idiopathic pericarditis and the benefit of corticosteroid treatment should be considered in SS patients.

In addition, there have been infrequent reports of arrhythmogenic mechanisms. In a study by Segreti et al., a Brugada syndrome was identified in a patient with SS (29). Similarly, Sharma et al. reported a case of overgrowth and dysmorphic features in a patient with Wolff Parkinson White Syndrome (WPWS) in 2003, even though no genetic findings were reported, and SS was not formally diagnosed (30). In our own cohort, we have also observed a comparable form of arrhythmia, suggesting a potential underlying mechanism that warrants further investigation in future studies.

The increased oncological risks afflicting SS patients are of great concern to geneticists, pediatricians, oncologists, and families. Somatic variants affecting *NSD1* have been identified in various types of tumors, suggesting a potential tumor suppressing role (31). Tauchmann et al. recently reviewed the role and molecular functions of *NSD1*, in which it is shown to be widely expressed in different tissues, playing an important role in human developmental syndromes, such as SS, as well as in different types of malignancies (32, 33).

Indeed, the genetic profile of SS has great repercussions on the clinical presentation of these patients. In our study, we found a correlation between oncological risk and missense *NSD1*. Future studies to understand the functional involvement of these variants and the role of *NSD1* would be of great interest. Most of the reported malignancies in SS have been solid tumors, such as Wilms tumor, hepatocarcinoma, neuroectodermal tumor, small cell lung carcinoma, teratomas, and neuroblastoma. However, other malignancies, including lymphoproliferative disorders such as acute lymphoblastic leukemia, B-cell lymphoblastic lymphoma, and other non-Hodgkin lymphomas, have also been reported (34, 35).

In our cohort, two patients presented solid tumors: one of them corresponds to a stable splenic hamartoma, and another one presented a retinal melanocytoma as a finding on ocular examination, asymptomatic at the time of diagnosis. None of these tumors had been previously described in SS.

Interestingly, although with less involvement in the severity of the disease but potentially very afflictive, five patients presented onychocryptosis, not previously associated with this pathological entity. Particular nail care may be useful to prevent this outstanding complication in our cohort, probably explained by distal phalanx or nail malformations or connective tissue anomalies.

Since it is difficult to determine if these atypical findings are sporadic or clearly secondary to SS, we recommend increasing awareness among physicians, annotating and reporting them. Associating these new phenotypes with the wider SS clinical spectrum should be considered.

Future descriptions are needed on this issue in order to expand the specific follow-up protocols. What does seem mandatory is the need for a multidisciplinary approach to patients with SS.

## 5. Conclusions

This study deepens in the already known phenotype but also considers multiple atypical findings that may have been not described but are probably frequent and relevant in SS. Remarkably recurrent pericarditis, onychocryptosis, and unusual malignancies were found in our large pediatric sample indicating that multidisciplinary and comprehensive follow-up recommendations are needed. The clinical and molecular spectrum of SS in this sample is described in detail, highlighting the importance of continuously revisiting and expanding the phenotype and genotype of rare diseases, as well as, exploring the possible correlations among them. Unfortunately, we were not able to establish a clear correlation between phenotype and genotype. However, we found that ASD is more frequently diagnosed in males than in females; contrarily feeding disorders are more prevalent in females. *NFIX* patients had more prevalent feeding disorders but fewer malignancies than *NSD1* patients. The findings of this study can aid in directing follow-up, screening for associated comorbidities, and assessing newly described health complications. Overall, this study emphasizes the need for continued research into rare diseases to better understand their clinical and molecular characteristics, revisit and redesign follow-up guidelines, and, as a final goal, improve patients' and families’ outcomes and wellbeing.

## Data Availability

The raw data supporting the conclusions of this article will be made available by the authors, without undue reservation.
