# Correction: Beyond the known phenotype of sotos syndrome: a 31-individuals cohort study

**DOI:** 10.3389/fped.2026.1880797

**Published:** 2026-06-02

**Authors:** Lourdes Vega-Hannah, Mario Sanz-Cuesta, Didac Casas-Alba, Mercé Bolasell, Loreto Martorell, Leticia Pías, Ana Lucia Feller, Antonio F. Martinez-Monseny, Mercedes Serrano

**Affiliations:** 1 Department of Pediatrics, Hospital Sant Joan de Déu Barcelona, Barcelona, Spain; 2 Department of Pediatrics, Hospital de Sant Boi, Parc Sanitari Sant Joan de Déu, Barcelona, Spain; 3 Department of Genetic and Molecular Medicine/IPER, Institut de Recerca, Hospital Sant Joan de Déu Barcelona, Barcelona, Spain; 4 Pediatric Neurology Department, Institut de Recerca, Hospital Sant Joan de Déu, Barcelona, Spain; 5 Departamen of Pediatrics, Hospital J P Garrahan, Buenos Aires, Argentine; 6 Centro de Investigación Biomédica en Red de Enfermedades Raras (CIBERER), Instituto de Salud Carlos III, Barcelona, Spain

**Keywords:** sotos syndrome, phenotype, overgrowth, *NSD1*, *NFIX*, *APC2*

Author name (inversion of names and surnames)

In the published article, the author names appeared incorrectly. The correct author list and names appear below.

Lourdes Vega-Hanna*, Mario Sanz-Cuesta*, Didac Casas-Alba, Mercè Bolasell, Loreto Martorell, Leticia Pías, Ana Lucia Feller, Antonio Federico Martínez-Monseny, Mercedes Serrano

Equal contribution statement

In the published article, the equal contribution statement was missing for authors Lourdes Vega-Hanna* and Mario Sanz-Cuesta*. The following statement has been included.

*These authors have contributed equally to this work

The original article has been updated.

