# Supplementary material for: Beyond the known phenotype of sotos syndrome: a 31-individuals cohort study
Source: Front Pediatr. 2023 Jun 13;11:1184529. doi: 10.3389/fped.2023.1184529 (PMC10298147; doi:10.3389/fped.2023.1184529)
Supplement: Supplementary file 1 [file Table1.docx]

Supplementary Material

Beyond the known phenotype of Sotos Syndrome: a 31-individuals cohort study

**Vega-Hanna Lourdes^1^, Sanz-Cuesta Mario^2^, Casas-Alba Didac^3,4^, Bolasell Mercè^3^, Martorell Loreto^3^, Pías Leticia^3,4^, Feller Ana Lucia^5^, Martínez-Monseny Antonio F^3^, Serrano Mercedes^4,6^.**

^1^Department of Pediatrics, Hospital Sant Joan de Déu Barcelona, Barcelona, Spain

^2^Department of Pediatrics, Hospital de Sant Boi, Parc Sanitari Sant Joan de Déu, Barcelona, Spain

^3^Department of Genetic and Molecular Medicine/IPER, Institut de Recerca, Hospital Sant Joan de Déu Barcelona, Barcelona, Spain

^4^Pediatric Neurology Department, Institut de Recerca, Hospital Sant Joan de Déu, Barcelona, Spain

^5^Departamen of Pediatrics, Hospital J P Garrahan, Buenos Aires, Argentine.

^6^Centro de Investigación Biomédica en Red de Enfermedades Raras (CIBERER), Instituto de Salud Carlos III, Barcelona, Spain

*** Correspondence:**Antonio Martínez-Monseny, MD, PhD.

afmartinez@sjdhospitalbarcelona.org

# Supplementary Table 1
